# Identification and Characterization of Key Genes Associated with Amelogenesis

**DOI:** 10.1055/s-0044-1787958

**Published:** 2024-09-19

**Authors:** Tahsinul Haque, Fatema Akhter, Nourelhoda Alim, Abdullah Nabhan, Fawzia Al kahtani, Abdullah Mohammed Sambawa

**Affiliations:** 1 Preventive Dental Sciences Department, College of Dentistry, Dar Al Uloom University, Riyadh, Saudi Arabia; 2 Surgical and Diagnostic Sciences Department, College of Dentistry, Dar Al Uloom University, Riyadh, Saudi Arabia; 3 Surgical and Diagnostic Sciences Department, College of Dentistry, Dar Al Uloom University, Riyadh, Saudi Arabia; 4 Surgical and Diagnostic Sciences Department, Collage of Dentistry, Prince Sattam Bin Abdulaziz University, Al Kharj, Saudi Arabia; 5 Dental Public Health, Private Sector, Riyadh, Saudi Arabia; 6 Primary Healthcare Center, Ministry of Health, Riyadh, Saudi Arabia

**Keywords:** amelogenesis, genes, genetics, preameloblasts, secretory ameloblasts, tooth development

## Abstract

**Objectives** The identification of key genes associated with amelogenesis would be helpful in finding solutions to genetic disorders in oral biology. The study aimed to use *in silico* analysis to identify the key genes involved in tooth development associated with preameloblasts (PABs) and secretory ameloblasts (SABs).

**Material and Methods** The data was subjected to quality analysis and uniform manifold approximation and projection analysis. To examine the distribution of the genes and identify important upregulated loci, a *p* -value histogram, a quantile plot, a mean difference and mean-variance plot, and a volcano plot were generated. Finally, protein-protein interaction and gene enrichment analyses were performed to determine the ontology, relevant biological processes, and molecular functions of selected genes.

**Results** A total of 157 genes were found to be significant in the PAB versus SAB comparison. HIST1H31 revealed strong interaction with HIST1H2BM, and EXO1, ASPM, SPC25, and TTK showed strong interactions with one other. The STRING database revealed that NCAPG, CENPU, NUSAP1, HIST1H2BM, and HIST1H31 are involved in biological processes. NCAPG, CENPU, SPC25, ETV5, TTK, ETV1, FAM9A, NUSAP1, HIST1H2BM, and HIST1H31 are involved in cellular components.

**Conclusion** The TTK, NUSAP1, CENPU, NCAPG, FAM9A, ASPM, SPC25, and HIST1H31 genes demonstrate functions in cell division. These genes might play a role in ameloblast development. These results will be useful in developing new methods to stimulate ameloblast development, which is essential for tooth regeneration and tissue engineering. However, more research is required to validate the functions of these genes and the genes with which they interact. A wide variety of genetic, epigenetic, and exogenous signaling factors regulate these genes and pathways throughout development and differentiation, cell fate, and behavior.

## Introduction

Amelogenesis is the process by which enamel is formed by epithelial ameloblasts facing the odontoblast layer. More advanced odontoblasts and stratum intermedium cells initiate ameloblast differentiation via molecular signals. Amelogenins, enamelins, ameloblastins, and tuftelins, which are released into the extracellular space by ameloblasts, are the major proteins of the enamel matrix. Enamel changes into highly mineralized tissue, and as a result, ameloblasts consume debris. 1 Apoptosis kills approximately half of the ameloblasts during amelogenesis, and the remaining half dies after the process has been completed. Amelogenesis is a process of gradual differentiation governed by a number of molecular and morphogenetic events and is distinguished by the formation of enamel and the differentiation of ameloblasts. Enamel is made by epithelial cells called ameloblasts that fuse with the odontoblast layer in a process called amelogenesis. The process of amelogenesis ends when the tooth erupts, and there is no secondary or regenerative enamel development afterward. Therefore, amelogenesis is distinguished by the transition from preameloblast (PAB) to secretory ameloblast (SAB). Following the disintegration of the basement membrane during the bell stage of tooth formation, 2 induced by signals from the underlying dental mesenchymal compartment, PABs differentiate into SABs, thereby initiating the differentiation process. These extracellular matrix proteins are necessary for the formation and mineralization of enamel, 3 and disruption of ameloblast protein production results in deformed enamel. 4 Ameloblasts differentiate into short mature ameloblasts (MABs) that regulate enamel mineralization after laying down a full-thickness enamel matrix. MABs die by apoptosis 5 following tooth eruption. Because adults lack easily available ameloblast-lineage cells, enamel cannot be restored or regenerated. Amelogenesis imperfecta (AI) is a collection of inherited diseases that cause changes in the structure and appearance of dental enamel and are frequently associated with changes in other intraoral or extraoral tissues. AI is a group of genetically determined disorders that have a relatively uniform effect on the structure and clinical appearance of the enamel on all or nearly all teeth and that can be linked to morphologic or biochemical abnormalities elsewhere in the body. 6 Amelogenin is expressed in humans and cattle from genes on the X and Y chromosomes. In human males, amelogenin (AMELX) transcripts are expressed from the X chromosomal copy of the gene, while 10% are expressed from the Y chromosomal copy (AMELY). The ENAM gene in this region has been identified as a potential gene affected in the form of AI. 7

The ENAM gene contains 10 exons and 9 introns. The ameloblastin (AMBN) gene is strongly expressed by ameloblasts and weakly expressed by odontoblasts and preodontoblasts, with moderate expression also observed in Hertwig's epithelial root sheath and odontogenic benign tumors with malignant behavior such as ameloblastoma. The gene encoding human enamelysin (matrix metalloproteinase-20, or MMP-20) resides on chromosome 11. Many studies have found that MMP-20 is only present in teeth. A genetic mutation of MMP-20 was discovered in two family members affected with autosomal-recessive pigmentary hypomaturity. This mutation removes the splice acceptor at the 3′ end of intron 6 and results in the production of a hypomature enamel product. The most likely outcomes appear faulty splicing with both defects introducing an upstream translation termination codon. 8 The KLK4 gene, found on chromosome 19, is a member of the human tissue kallikrein gene family. KLK4 is involved in fragmenting smaller pieces of the tumor necrosis factor receptor-associated protein amelogenin cleavage product. In the case of KLK4, one mutation leads to a shortened protein that has 152 but lacks the S207 site, which is required for the enzyme to function; because of this aberrant enzyme activity, the enamel crystallites expand to standard length but only partial thickness. 9

Amelogenesis is one of the important mechanisms in tooth development. Unraveling the role of genes associated with the mechanisms will open new avenues for diagnostic techniques and cures related to tooth disorders. *In silico* studies in which gene identification and functional genomics studies are performed before designing follow-up laboratory research, are very useful. In the present study, various bioinformatics tools were used to identify and characterize amelogenesis-related genes to unravel their roles in tooth development. Moreover, using bioinformatics tools to study genes associated with dental biology and health is frequent in the dental sciences. Identification of relevant molecules and signaling networks with greater precision will shed light on the mechanisms that govern enamel formation and lay the groundwork for elucidating the mechanisms that regulate the differentiation of PABs into SABs to better understand enamel regeneration. This study has provided new information that will be helpful in finding pragmatic treatments for genetic disorders associated with amelogenesis.

## Materials and Methods

### Data Mining and Quality Control Analysis

The data was retrieved from the Gene Expression Omnibus (GEO) data set series GSE59214. After collecting expression profiling using array data, the GEO2R Web tool was used for further analysis to identify genes differing in expression across various experimental conditions. GEO2R is an interactive resource for contrasting two or more sample groups within a GEO series to locate genes that are expressed differentially in differing experimental settings.

The findings have been presented in the form of a gene table in order of importance, a series of graphical plots to aid in the visualization of differentially expressed genes, and a quality assessment of the data set, using the Bioconductor project's GEO query 10 and limma R packages. 11 Limma (linear models for microarray analysis) is a popular statistical technique for identifying differentially expressed genes using the R programming language. Statistical *p* -values were subjected to multiple-testing corrections in a wide range of experimental designs and data sources to reduce the likelihood of false-positive results. For comparison, the data was divided into two groups, namely, PAB and SAB, and each group contained three replications. To normalize data 12 and visualize the distribution of the values in the selected samples, an expression box plot and a density plot were generated. 13

### Uniform Manifold Approximation and Projection Analysis

Next, a uniform manifold approximation and projection (UMAP) plot was generated. UMAP is a technique of dimension reduction that can be used to better view the connections that exist between individual samples. 14

### Value Histogram

To analyze the distribution of all genes according to their *p* -values, a *p* -value histogram plot 15 was generated.

### Mean Difference and Mean-Variance Plots

A mean difference plot was constructed to display the results of a single comparison (one group of PABs compared to another group of SABs). A mean-variance plot was generated to analyze the variation in the data. 16

### Volcano Plot

To identify the top-upregulated genes, a volcano plot 17 analysis was employed. The top 20 upregulated genes were selected according to the lowest adjusted *p* -values. The most dependable genes exhibited the lowest *p* -values ( Table 1 ).

**Table 1:** Top upregulated genes

ID	Adjusted *p* -value	*p* -Value	Log FC	Gene symbol	Gene title
8151341	0.00178	0.00000010 7	4.357	TRPA1	Transient receptor potential cation channel subfamily A member 1
8138289	0.00584	0.00000114	3.912	ETV1	ETS variant 1
8056572	0.00584	0.00000158	3.749	SPC25	SPC25, NDC80 kinetochore complex component
7940147	0.00584	0.00000138	3.551	FAM111B	Family with sequence similarity 111 member B
7923086	0.00584	0.00000082 5	3.218	ASPM	Abnormal spindle microtubule assembly
7967993	0.00584	0.00000105	2.564	FGF9	Fibroblast growth factor 9
8124531	0.00584	0.00000151	2.367	HIST1H3I	Histone cluster 1, H3i
8117594	0.01494	0.0000056	3.903	HIST1H2BM	Histone cluster 1, H2bm
7909946	0.01494	0.00000844	3.592	FAM177B	Family with sequence similarity 177 member B
8052355	0.01494	0.00000775	3.241	EFEMP1	EGF containing fibulin-like extracellular matrix protein 1
8171260	0.01494	0.00000886	3.189	FAM9A	Family with sequence similarity 9 member A
8094278	0.01494	0.00000679	2.939	NCAPG	Non-SMC condensin I complex subunit G
8103932	0.01494	0.00000783	2.862	CENPU	Centromere protein U
7926728	0.01494	0.00000823	2.285	MYO3A	Myosin IIIA
7910997	0.01494	0.00000791	2.149	EXO1	exonuclease 1
8117426	0.01494	0.0000085	1.94	HIST1H2BH///HIST1H2B H	Histone cluster 1, H2bh///histone cluster 1, H2bh
8101788	0.01528	0.00000964	2.355	UNC5C	unc-5 netrin receptor C
7982889	0.01651	0.0000109	3.443	NUSAP1	Nucleolar and spindle associated protein 1
8120838	0.01659	0.0000118	2.563	TTK	TTK protein kinase

Abbreviations: EGF, epidermal growth factor; ETS, erythroblast transformation-specific; FC, fold change; SMC, structural maintenance of chromosomes.

Note: The top 20 upregulated genes, selected according to the lowest adjusted *p* -values (< 0.05).

### Protein-Protein Interaction and Gene Enrichment Analysis

An investigation of protein-protein interactions 18 was performed on the selected genotypes. The g:Profiler web was used for gene enrichment analysis 19 to analyze the ontology, relevant biological processes, and molecular functions of selected genes.

## Results

### Data Normalization Quality Control

**Fig. 1:**
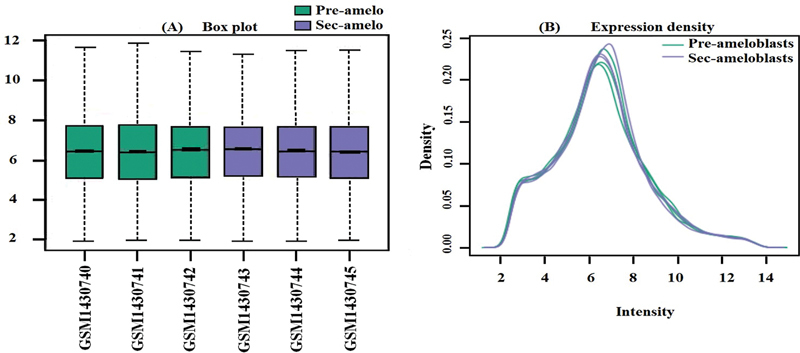
( **A** ) Diagnostic plots for microarray data from three samples of preameloblasts and three samples of secretory ameloblasts. ( **B** ) Density plot of preameloblast and secretory ameloblast samples.

Because the expression data was normalized, all selected samples had the same value distribution. The box plots in Fig. 1A show the normalization of two samples. The first sample represents PABs with three replications, and the second sample represents SABs with three replications. The plots show the data variation between the PABs and the SABs with their replications. The density expression plot in Fig. 1B shows that the variation between the PABs and the SABs was small.

### UMAP and *p* -Value Histogram Plot

**Fig. 2:**
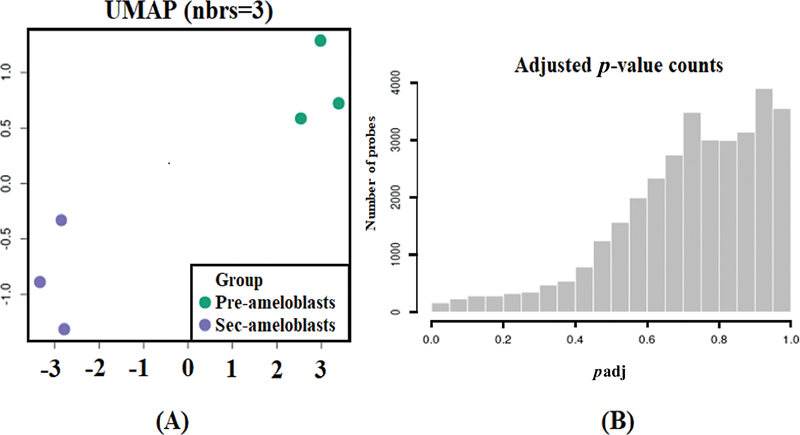
( **A** ) Uniform manifold approximation and projection (UMAP) plot generated from three samples of preameloblasts and three samples of secretory ameloblasts. ( **B** ) Adjusted *p* -value histogram showing the *p* -value distribution for all analyzed genes.

The plot in Fig. 2A shows that there were substantial differences among thousands of genes in PABs and SABs. Variations among three biological replicates of PABs and three biological replicates of SABs were minimal; however, a distinct difference between samples of PABs and SABs was noted. The histogram in Fig. 2B displays the set of *p* -values. From the *p* -value histogram, it is evident that the *p* -values were not uniformly distributed.

### Variance Trend and Mean Difference Plot

**Fig. 3:**
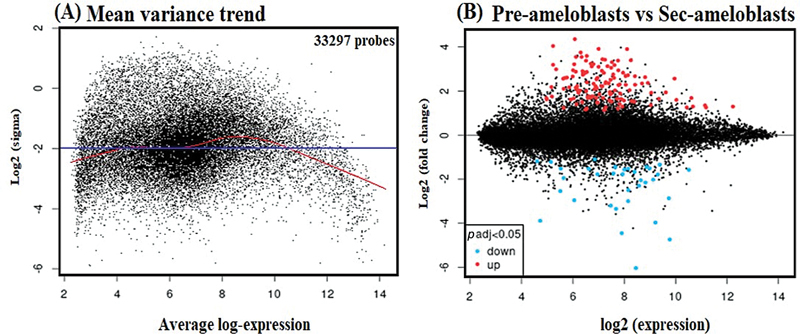
( **A** ) Variance trend plot of preameloblasts versus secretory ameloblasts. Each dot corresponds to a gene. The red line represents an approximation of the mean-variance trend. The blue line is an approximation to a constant variance. ( **B** ) Mean difference plot of preameloblasts versus secretory ameloblasts.

The graph shown in Fig. 3A illustrates the relationship between the mean and variance of the PAB and SAB expression data and aided in determining whether there was a high level of variety in the data. This graph could also be used to determine whether the precision weights option for accounting for mean-variance trends should be used; precision weights improve test result precision when there is a strong mean-variance trend. Each dot corresponds to a gene. The mean difference plot of PABs versus SABs denotes upregulated genes by red dots and downregulated genes by blue dots, showing the average of normalized counts versus log2 fold changes for all genes studied.

### Venn Diagram

**Fig. 4:**
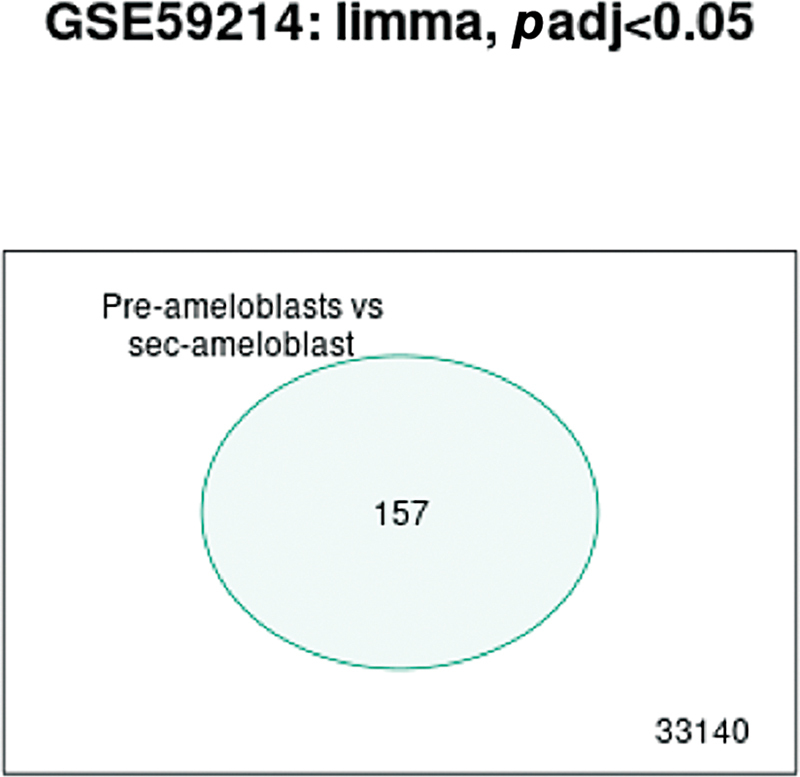
Venn diagram of preameloblasts versus secretory ameloblasts.

A Venn diagram of the genes indicated that out of a total of 33,140 genes, 157 were significant in the PAB versus SAB comparison ( Fig. 4 ). Data regarding the significant genes has been provided in the Excels spreadsheet.

### Volcano Plot

**Fig. 5:**
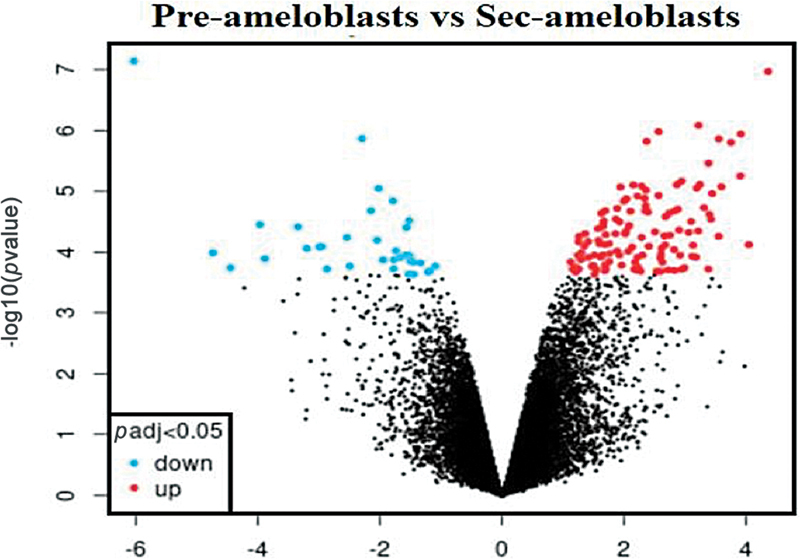
Volcano plot of upregulated and downregulated genes in preameloblasts versus secretory ameloblasts.

The volcano plot ( Fig. 5 ) of PABs versus SABs presents *p* -value versus magnitude of change statistical significance (fold change). It represents the identification of genes with large, statistically significant changes. In this volcano plot, the most upregulated genes are on the right, the most downregulated genes are on the left, and the most statistically significant genes are at the top.

The volcano plot was constructed to identify the top-upregulated genes in PABs versus SABs. The increase in upregulated genes was fourfold in the PAB versus SAB plot. All of the gene data is presented in an Excel spreadsheet.

### Protein-Protein Interactions

**Fig. 6:**
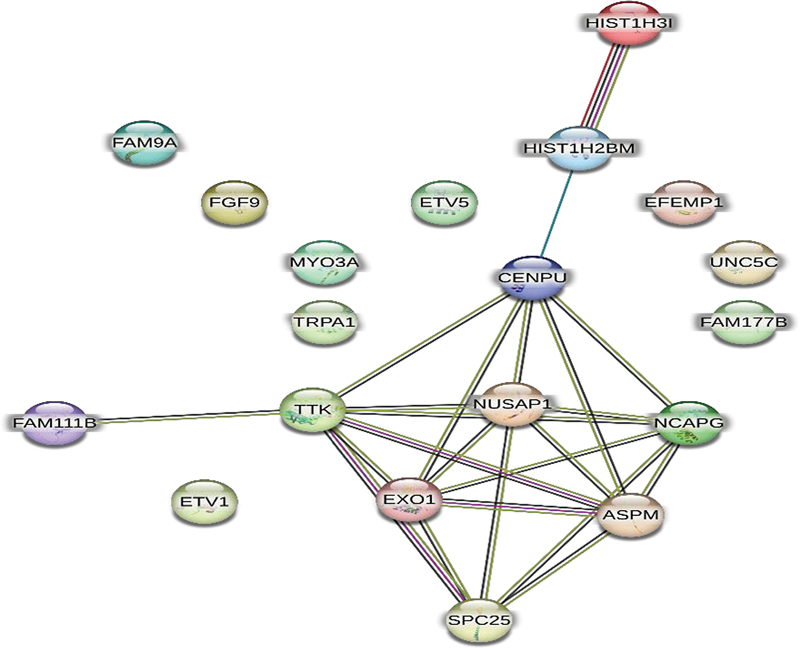
Protein-protein interaction analysis.

Protein-protein interaction analysis was performed on the set of genes selected according to the adjusted *p* -values. According to this analysis, HIST1H31 revealed strong interaction with HIST1H2BM, and EXO1, ASPM, SPC25, and TTK showed strong interaction with one other ( Fig. 6 ). The STRING database indicates that NCAPG, CENPU, NUSAP1, HIST1H2BM, and HIST1H3I are involved in biological processes. NCAPG, CENPU, SPC25, ETV5, TTK, ETV1, FAM9A, NUSAP1, HIST1H2BM, and HIST1H3I are related to cellular components ( Table 1 ).

### Gene Enrichment Analysis

**Fig. 7:**
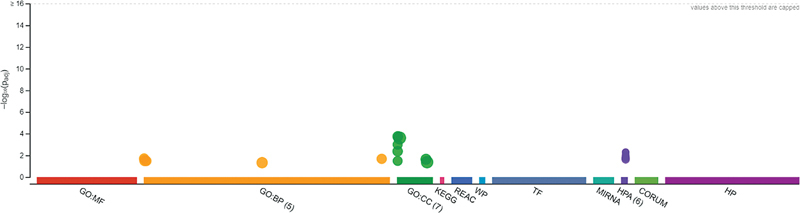
Functional enrichment analysis graph.

**Fig. 8:**
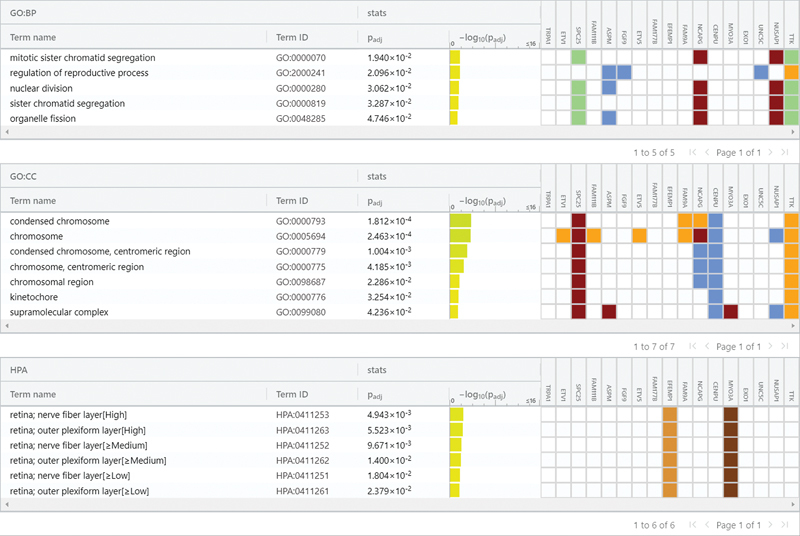
Table of gene ontology with terms, term identifiers (IDs), and *p* -values.

Functional enrichment analysis was performed on selected genes from PABs and SABs. This analysis provided information regarding the molecular function of gene ontology, biological process of gene ontology, and cellular components of gene ontology. The analysis revealed that the genes are involved in biological processes, cellular components, and KEGG pathways, as shown in Fig. 7 . The table of gene ontology with *p* -value is shown in Fig. 8 , and the functional descriptions are provided in the Excel data sheet.

## Discussion

Ameloblasts are a type of cells responsible for the deposition of enamel and the production of extracellular matrix proteins. 3 SABs deposit extracellular matrix, and the MABs mineralize the organic matrix, resulting in the production of enamel, 20 one of the hardest mineralized tissues in vertebrates. 21 Three stages of ameloblast differentiation together produce a fully developed ameloblast: the presecretory, secretary, and maturation stages. 2 Terminally developed cells called squamous ameloblasts and molar ameloblasts are formed from undifferentiated ameloblasts called PABs during odontogenesis. 22 PABs are dental epithelial cells that are elongated and columnar in shape, with their nuclei clustered along the stellate reticulum as well as their cytoplasm, which contains the organelles required for protein synthesis and secretion of enamel. Enamel formation and ameloblast differentiation are hallmarks of the process of amelogenesis, which is governed by a number of molecular and morphogenetic events. It is generally agreed that one of the defining characteristics of amelogenesis is the change from PAB to SAB. The niche in which ameloblasts differentiate is composed of secreted proteins, enzymes, signaling molecules, and other components.

The transition from PAB to postameloblast occurs during ameloblast differentiation, and it has a profound effect on the secretion of matrix proteins in the enamel and the commencement of mineralization. Signaling molecules, their receptors, and transcriptional regulators all work together to control this process. 22 However, the transcriptional pathways governing ameloblast development remain poorly understood. Specifically, we sought to identify genes that upregulate during the ameloblast transition from PAB to SAB. According to a study by Liu et al, 23 four genes (AMBN, AMTN, ENAM, and MMP-20) are associated with SABs, consistent with previous studies. They revealed that cell cycle control, deoxyribonucleic acid (DNA) damage repair, and apoptosis were linked to high-expression genes in PABs among other differentially expressed genes. On the other hand, highly expressed genes in SABs were linked to cell adhesion and extracellular matrix. 23 Another study revealed that SABs released extracellular matrix proteins into the enamel matrix consisting of three structural proteins (AMELX, AMBN, and ENAM). MMP-20 and KLK4 separate these proteins from the matrix in phases during the ameloblast secretory and maturation processes, respectively. 22

Real-time polymerase chain reaction (PCR) validated AMELX enrichment in SABs, whereas microarray analysis did not. Microarrays have a lower detection limit as opposed to real-time PCR; thus, the fold changes seen by microarrays are typically smaller. 24 Additional gene enrichment analysis of SABs revealed the presence of five genes (DSPP, DMP1, PHEX, ALPL, and MMP-16) involved in biomineralized tissue production, all of which have the potential to control ameloblast differentiation and enamel formation.

In this study, an analysis of gene enrichment was performed in order to conduct a comprehensive study of the genes. In the gene enrichment study, it was discovered that of the selected genes, TTK, NUSAP1, HIST1H2BM, NCAPG, CENPU, FAM9A, SPC25, ETV5, ETV1, MYO3A, EFEMP1, and HIST1H3I are engaged in biological processes such as regulation of reproductive processes, nuclear division, and mitotic sister chromatid segregation. They are also present in cellular components, condensed chromosomes, centromeric regions, kinetochores, supramolecular complexes, and chromosomal regions. When compared to SABs, PABs have higher expression data. The genes TTK, NUSAP1, CENPU, NCAPG, FAM9A, ASPM, SPC25, and HIST1H3I function in mitotic sister chromatid segregation, cell division, and organelle fission. In comparisons of conditions of PABs and SABs, the *p* -values of these genes were lower than 0.05.

## Conclusion

We compared two groups, namely, PABs and SABs. During tooth development, the expression of genes involved in the regulation of the cell cycle, repair of DNA damage, and apoptosis pathways regulate PAB maturation. Several signaling mechanisms govern SAB cell behavior, specifically the production of enamel matrix proteins and cell adhesion, both of which are required for enamel development and cell-cell interactions. PAB versus SAB expression data was used to study gene function. The TTK, NUSAP1, CENPU, NCAPG, FAM9A, ASPM, SPC25, and HIST1H3I genes function in cell division, and these genes may play a role in ameloblast development. These results will be useful in developing new methods to stimulate ameloblast development, which is essential for tooth regeneration and tissue engineering. However, further research is required to validate the functions of these genes and the genes with which they interact. A wide variety of genetic, epigenetic, and exogenous signaling factors regulate these genes and pathways to direct throughout development and differentiation, cell fate, and behavior.
